# Evaluation of the antitumor activity of moronecidin (Piscidin)-like peptide in combination with anti-PD-1 antibody against melanoma tumor

**DOI:** 10.22038/IJBMS.2023.69639.15166

**Published:** 2023

**Authors:** Mohsen Mohammadi, Amin Moradi Hasan-Abad, Ali Ghasemi

**Affiliations:** 1 The Persian Gulf Marine Biotechnology Research Center, The Persian Gulf Biomedical Sciences Research Institute, Bushehr University of Medical Sciences, Bushehr, Iran; 2 Autoimmune Diseases Research Center, Shahid Beheshti Hospital, Kashan University of Medical Sciences, Kashan, Iran; 3 Department of Biochemistry and Hematology, Faculty of Medicine, Semnan University of Medical Sciences, Semnan Iran; 4 Cancer Research Center, Semnan University of Medical Sciences, Semnan, Iran

**Keywords:** Anti-PD-1, Antibody, Antimicrobial peptides, Cancer therapy, Immunotherapy, Melanoma cancer

## Abstract

**Objective(s):**

Immunotherapy has changed the landscape of oncology over the last decade and has become a standard of care for various cancers. Researchers previously demonstrated that B16-F10 melanoma in C57Bl6 mice is resistant to immune checkpoint inhibitors. The goal of this study was to investigate how anti-PD1 antibodies functioned in combination with a new antimicrobial peptide (AMP) called moronecidin-like peptide (MLP).

**Materials and Methods:**

We studied the cytotoxic effect of AMP on the B10-F16 tumor cell line with the MTT experiment. The necrotic and apoptotic cells were determined by Presidium iodide (PI) /Annexin V staining and flow cytometry-based methods. Mice were inoculated subcutaneously with B10-F16 tumor cells in the mammary gland. Each group was sacrificed two weeks after the last injection to examine tumor-specific CD8+ T cell responses using flow cytometry.

**Results:**

Annexin V and PI staining assay revealed that MPL significantly induces apoptosis in B16F10 cells. It should be noted that MLP in combination with anti-PD-1 improved antigen-specific T-cell responses synergistically (*P*=0.01) when compared with respective monotherapy. Furthermore, when compared with the respective monotherapies, combination therapy significantly controlled tumor growth in B10-F16 tumor cells and increased survival rate.

**Conclusion:**

Treatments with anti-PD-1 inhibitors alone had only a minor effect on tumor size, whereas combination therapy resulted in significant tumor growth control and increased animal survival. MLP therapy combined with anti-PD-1 antibody improves anti-tumor immune response in addition to inducing tumor cell apoptosis. As a result, the evidence suggests that intratumoral injection of MPL can improve anti-PD-1 antibody antitumor response.

## Introduction

Melanoma accounts for approximately 1.7 % of all new cancer cases and 0.7 % of all cancer deaths worldwide each year (1). Treatment options for malignant melanoma may include tumor resection surgery, immunotherapy, gene therapy, and chemotherapy. Each of these methods has many disadvantages and toxic side effects (2). Also, cancer is still the main cause of death in the world. The successful treatment of some types of cancer with immunotherapy has strongly encouraged researchers to use it alongside conventional treatments to increase effectiveness (3). 

The principal goal of cancer immunotherapy is to harness immune responses (particularly CD8 T cell responses) against cancer, so the induction of tumor-specific CD8 T cell responses is a hallmark of successful cancer immunotherapy (4). *In situ* vaccination, intratumoral injection of immunostimulatory reagent, is a promising strategy for priming tumor-specific immune response without prior knowledge of tumor antigens, capable of inducing a broad T cell response without the side effects associated with systemic immunomodulatory administration (5). Nonetheless, the therapeutic effect of the tumor-specific CD8 T cell response could be affected by immune checkpoint receptors (i.e., programmed cell death protein 1 (PD-L1) and B and T-lymphocyte attenuator (BTLA) that send inhibitory signals to T cells) (6). In this regard, it has been demonstrated that blocking PD-1/PD-L1 interaction with an anti-PD-1 antibody is beneficial for the treatment of various types of cancer (particularly in the case of melanoma) (7, 8). One of the most important ICIs is the anti-PD-1 antibody, which has been used in the treatment of various cancers such as bladder, liver, and lung, with promising results in some of these studies (9). The promising therapeutic outcomes of using immune checkpoint inhibitors in clinics have opened a new and hopefully promising window for cancer treatment (3). However, the low response rate (10 to 30%) of ICIs reagents against various cancer types limits their administration to all patients. Given that the therapeutic effect of anti-PD-1 antibodies depends on the frequency of tumor-infiltrating CD8 T cells (8), combining anti-PD-1 antibodies with the immunostimulatory agents that induce tumor-specific CD8 T cells may be a sensible strategy for improving anti-PD-1 antibody therapeutic effect. Antimicrobial peptides (AMPs), which are small molecular weight peptides (MW5kDa) that play an important role in innate immunity, possess the appropriate properties that make them effective immunostimulatory agents for intratumoral injection. Actually, AMPs possess both direct and indirect anti-cancer effects by inducing apoptosis in tumor cells, which results in tumor antigen release, and immunostimulatory effects by activating dendritic cells (DCs) (10). These peptides specifically recognize and induce apoptosis in tumor cells by binding to negatively charged moieties on the extracellular surface of tumor cell membranes, such as phosphatidylserine (11). Also, it has been reported that intratumoral injection of AMPs stimulates tumor-specific T-cell responses (12). Piscidin-1 (moronecidine) is a well-known cationic AMP and a member of the piscidin family produced and secreted by hybrid striped bass (13). Although this peptide has been shown to be active against several human cancer cell lines, its cytotoxic function against normal cells (e.g., red blood cells) has limited its clinical use (14). In a recent study, a new AMP from the Piscidin family called moronecidine-like peptide (MLP) derived from the hippocampus was discovered; it has cytotoxic functions against tumor cells, meanwhile without hemolytic and cytotoxic activity against normal cells (15). As a result, MLP may be an effective small-molecule agent that can be used to treat cancer without raising concerns about off-target effects (13). 

The current study sought to determine whether intratumoral injection of MLPs could improve the anti-tumor immune response of anti-PD-1 antibodies in B10-F16 tumor-bearing mice.

## Materials and Methods

***Materials, cell line, and animals***

All chemicals were purchased from Sigma-Aldrich (Missouri, USA). The Melanoma cancer cell line (B10-F16) was purchased from the Pasteur Institute of Iran. Cells were cultured in Roswell Park Memorial Institute (RPMI) 1640 medium (Thermo Fisher Scientific, Waltham, MA, USA) containing fetal bovine serum (Thermo Fisher Scientific) and penicillin-streptomycin (Sigma-Aldrich, St Louis, MO, USA) in an incubator at 37 °C in 5% CO_2_. In our laboratory, pathogen-free female C57BL/6 mice aged between 6–9 weeks and weighing approximately 20–30 g have been tested and cared for under sterile conditions. Mice were purchased from the Karaj Laboratory Animal Centre of the Pasteur Institute of Iran. All experiments were conducted in accordance with the guidelines of the Ethical Committee of Pasteur Institute of Iran (Ethics number: IR.SEMUMS.REC.1400.041) and were approved by the NIH Guides for the Care and Use of Laboratory Animals. Annexin V/Propidium Iodide (PI) (Molecular Probes, A13201) apoptosis assay was used for the assessment of cellular apoptosis. APC antimouse CD3 antibody (Biolegend, UK), FITC anti-mouse CD8 antibody (Biolegend, UK), and PE anti-mouse IFN-γ antibody (Biolegend, UK) were used for tumor-specific CD8+ T cell staining. 

***Synthesis, purification, and identification of peptides***

Peptides were synthesized by PEPMIC Co., Ltd. using Fmoc solid-phase synthesis and HPLC purification. Peptide identity was confirmed by mass spectrometry. Each peptide was synthesized in quantities of 20 mg, and the peptide purity (as determined by RP-HPLC) was greater than 95%. The physicochemical properties, as well as the sequences of all peptides, have already been determined in various studies (15). 

***MLP cytotoxicity assessment on cancer cell line***

The cytotoxic effect was evaluated with the MTT experiment as described elsewhere (15). Briefly, B10-F16 (mouse melanoma cells) were cultured in 96-well plates at a concentration of 0.5 × 10^5^ cells per well. RPMI1640 medium containing 2 mM glutamine, 100 U/ml FBS 10%, penicillin, and 100 μg/ml streptomycin were used for cell culture; cells were incubated at 37 °C and 5% CO_2_ to reach 80% confluency. Then 100 μl of MLP or E7-derived peptide as non-AMP were added to the cells at 20, 40, 80, and 160 μM, and plates were incubated at 37 °C and 5% CO_2_ for 24 hr. Afterward, 20 μl of MTT reagent (5 mg/ml) was added to each well, and the plates were incubated at 37 °C for 4 hr. The culture medium was removed and 100 μl of DMSO was added to each well and the absorbance of each well was measured in 570 nm wavelengths with a microplate reader. Each experiment was repeated three times, and the cell proliferation inhibition rate was calculated according to the {(At-Ab)/(Ac-Ab)} × 100, formula, where, At is the absorbance value of the cells treated with various concentrations of nanoparticles, Ab is the absorbance value of the blank, and Ac is the absorbance value of cells without nanoparticles. 

***Assessment of apoptosis***

The necrotic and apoptotic cells are determined by Presidium iodide (PI) /Annexin V staining and flow cytometry-based methods. One of the early events of apoptosis is membrane phosphatidylserine (PS) translocation to the outside of the cell membrane. Annexin V has an affinity for PS, and fluorochrome-labeled Annexin V can be used for the detection of apoptosis by flow cytometry. PI is a membrane-impermeant dye that is generally excluded from viable cells. In dead cells, PI binds to DNA but PI cannot distinguish between apoptotic and necrotic cell deaths. After treatment of B10-F16 with MLP and non-AMPs for 4 hr, the cells were trypsinized, washed three times with serum-containing media, and resuspended in 400 μl of binding buffer. Four microliters of annexin V-FITC and 4 μl of PI stocking solutions were added to the suspension and the cells were incubated at room temperature in the dark for 5 min. Apoptotic cells were analyzed on flow cytometry (BD Biosciences), and the obtained data were analyzed using FlowJo software 7.6.1.

***Western blot analysis***

B10-F16 were cultured for 24 hr. The synthetic peptides with a final concentration of 20, 40, 80, and 160 μM were added and the cells were incubated further. Adhered cells were collected at 24, 48, and 72 hr after treatment. Proteins in cell homogenates were denatured for 5 min in a sample buffer before being fractionated by electrophoresis on a 12 % (w/v) gel. After transferring the protein bands to a PVDF membrane, the blots were blocked with 1X TBS, 0.1% Tween-20, and 5% BSA. After washing in TTBS, the blots were incubated for 1 hr with 1:200 dilutions of anti-Cyto-c and anti-Casp-3 biotinylated monoclonal antibodies, followed by HRP-labeled streptavidin. The complex was created by incorporating the horseradish peroxidase substrate diaminobenzidine (DAB, Roche, Germany). A rabbit anti-human actin antibody was used as a housekeeping control. 

***Tumor inoculation and evaluation of tumor volume and survival***

C57BL/6 mice were inoculated subcutaneously with B10-F16 tumor cells in the mammary gland. By injecting B10-F16 cells (5 × 10^5^ cells/ mouse) into mice, a primary tumor can be inoculated. When the initial volume of the tumors reached about 65 mm^3^, the mice carrying the B10-F16 tumor-bearing mice were randomly divided into 4 groups (5 mice per group) and treated as follows: in group I (control group), mice were treated intratumorally by phosphate-buffered saline (PBS) on days 1, 3, and 5. In group II, mice were treated intratumorally with MLP (500 μg/mouse) on days 1, 3, and 5 after tumor inoculation. In group III, mice were treated with anti-PD-1 antibodies (5 μg/mouse) intratumorally on days 1, 3, and 5 after tumor inoculation. In group IV, mice were treated with MLP (500 μg/mouse) plus anti-PD-1 (5 μg/mouse) antibodies intravenously on days 1, 3, and 5 after tumor inoculation. Two weeks after the last injection, each group was sacrificed to examine tumor-specific CD8 T cell responses by flow cytometry. The rest of the mice were kept for 30 days and the tumor size was measured and recorded every 2 days with a special ruler and calculated with the following ratio: Tumor volume (mm3) = 1/2 (length × width^2^) and at the final examination day (up to 32 days) tumors volumes were evaluated to determine survival. Mice were monitored until they reached their maximum size (approximately 1500 mm^3^), and mice that reach this size were killed.

***Tumor-specific CD8+ T cell responses***

The specific cytotoxic CD8+ T cell response was determined as described elsewhere (5). Briefly, two weeks after the last injection on day 5, each mouse in the four groups described above was sacrificed to examine tumor-specific CD8 T cell responses by flow cytometry. The spleen cells of the mice were harvested, and a single-cell suspension from the spleen cells was obtained by gently squashing them with a syringe plunger and 10 ml of PBS solution in a Petri dish, then centrifuged at 300×*g* to collect cell pellets. The cell pellets were then washed 3 times with PBS and after resuspension cultured on a 96-well plate at 5×10^5^/200 ml/well. The cells were then stimulated with B10-F16 cell protein lysate at 20 μg/ml and after cell treatment with monensin (1: 1000), the cells were incubated at 37 °C for 12 hr. The cells were stained using APC antimouse CD3 antibody and FITC anti-mouse CD8 antibody. For intracellular staining (ICS) the lymphocytes, the cells were fixed, permeabilized, and stained with PE anti-mouse IFN-γ antibody to determine CD8+ T lymphocytes expressing IFN-γ. The Percentage of the cells stained by anti-CD3+, CD8+, and IFN-γ was determined using flow cytometry, and the obtained data were analyzed using FlowJo software 7.6.1.

***Statistical analysis***

Data analysis was performed using the GraphPad PrismTM software (Version 6.02; San Diego, CA, USA). In addition, the data are presented as the mean ±standard deviation (SD). Statistical differences were analyzed by one-way Analysis of Variance (ANOVA). The results were conducted after at least three determinations for each test from three independent experiments. *P*-values<0.05 were statistically considered significant. The percentage of the cells stained by anti-CD3+, CD8+, and IFN-γ was determined using flow cytometry, and the obtained data were analyzed using FlowJo software 7.6.1.

**Figure 1:**
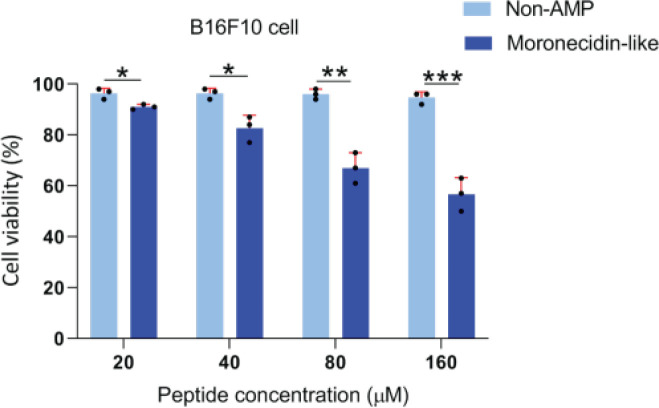
Graphical representation of the MTT test of melanoma cells after incubation with different concentrations of peptides

Effect of non-AMP and MLP on cell toxicity. Dose-dependent cytotoxicity of MLP on B16F10 cells over a 24 hr period using the MTT assay. The data showed that MLP has potent anticancer activities against cancer cell lines with respect to the control. The results shown are those of an experiment performed in triplicate. *P<0.05, **P<0.01, and ***P<0.001

**Figure 2:**
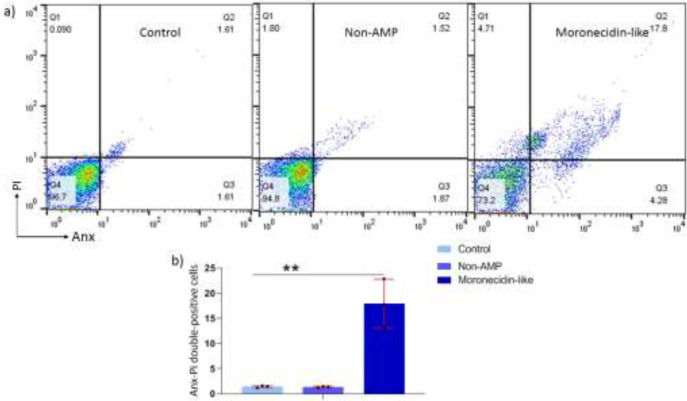
Apoptosis assay of tumor cells after exposure to MLP

a) Flow cytometry analysis of B16F10 cell lines treated with MLP for 24tide for 24eptide for 24Annexin-V-FITC/PI. Each experiment was repeated three times independently and the proportion of early apoptotic cells (Q3 area), later apoptotic cells (Q2 area), and necrotic cells (Q1 area) were measured in the one-step staining procedure. b) Results showed abundance of apoptosis in B16F10 cell lines treated with MLP. **P<0.01

MLP: Moronecidin-like peptide

**Figure 3:**
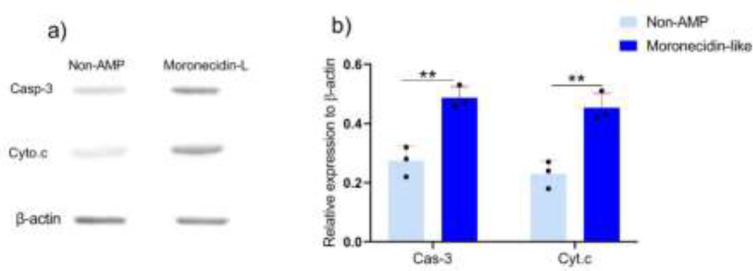
Apoptosis-related proteins were detected by Western blot

a) Western blotting showed changes in the abundance of apoptosis-related proteins (Cyto-c and Casp-3) in B16F10 cells treated with MLP for 24 hr. The spot band was analyzed using Image J software. Data were normalized using the β-actin level and presented as control normalization. b) The data (mean±SEM) represent at least 3 independent experiments. Significance was determined by Student's t-test. **P<0.01, comparison between the two groups

**Figure 4:**
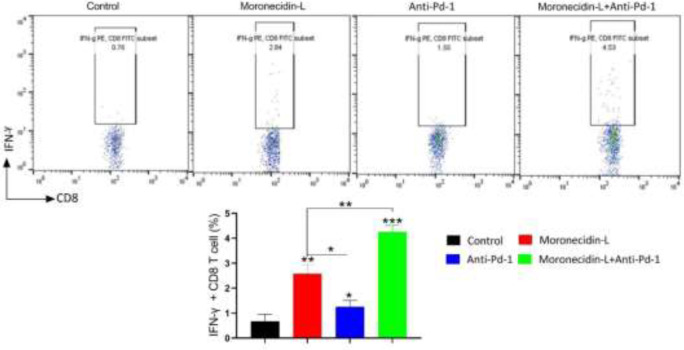
Flow cytometric analysis for tumor-specific CD8 T cell responses

IFN-γ-positive T cells in splenocytes were detected in mice treated with the AMPs and anti-PD-1 antibodies. Splenocytes were collected from mice 14 days after treatment. Production of IFN-γ in activated CD48 T cells was analyzed by flow cytometry. Representative flow cytometric results are presented (Top panel) and the percentile of cytokine-positive cells among the activated T cell subsets are summarized (bottom diagram) in all groups. Data represent mean+SD from each mice group. * *P*<0.01, **P<0.001, and ***P<0.001

**Figure 5.**
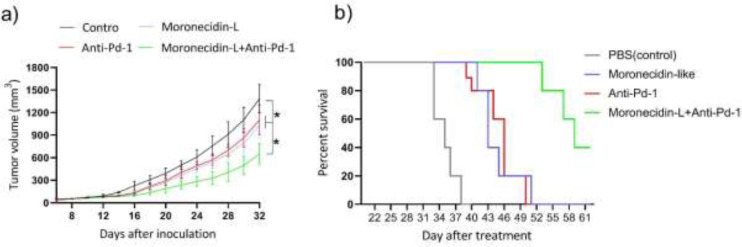
Tumor volume and survival curves after treatment in the tumor mouse model

Mice were inoculated subcutaneously with B10-F16 tumor cells in the mammary gland. Treatment was performed when the initial volume of the tumors reached about 65 mm2, and then the growth of the tumors was observed. In group I (control group), mice were treated intratumorally by PBS on days 1, 3, and 5. In group II, mice were treated intratumorally with MLP (500 μg/mouse) on days 1, 3, and 5 after tumor inoculation. In group III, mice were treated with anti-PD-1 antibodies (5 μg/mouse) intratumorally on days 1, 3, and 5 after tumor inoculation. In group IV, mice were treated with MLP (500 μg/mouse) plus anti-PD-1 (5 μg/mouse) antibodies intravenously on days 1, 3, and 5 after tumor inoculation. (a) Tumor volume curves of mice in the control, anti-PD-1, MLP, and MLP + anti-PD-1 groups after tumor inoculation. (b) Kaplan-Meier survival curves of mice receiving tumor inoculation. The significance values for the difference between the survival curves are Control vs anti-PD1: *P*<0.005, Control vs MLP: *P*<0.00001, control vs MLP + anti-PD1: *P*<0.00001, anti-PD-1 vs MLP: *P*<0.005, anti-PD1 vs MLP + anti-PD1:* P*<0.00002

## Results

***MLP cytotoxicity in the melanoma cell line***

MTT assay was used to determine cell cytotoxicity in the presence of MLP in the B16F10 cell line. MLP treatment showed that this peptide has potent anticancer activities against B16F10 cells in a dose-dependent manner. After 24 hr of treatment with the highest concentration of MLP (160 µM) B16F10 cells had the lowest cell proliferation compared with other concentrations (Figure 1). These findings suggested that MLP inhibited cell proliferation in this type of cancer cell at specific concentrations.

***Induction of apoptosis in the melanoma cell line after exposure to MLP***

Cells were stained with Annexin-V-FITC/PI to see if MLP caused apoptotic cell death. Annexin V and PI staining assay revealed that the proportion of B16F10 cells distributed in the areas of early apoptosis (Annexin V positive only, Q3 area) and later apoptosis (Annexin V and PI double positive, Q2 area) after MLP exposure was higher than in non-AMP peptides (Figure 2).

***Western blot analysis confirmed MLP-induced apoptosis in the melanoma cell line***

B10-F16 cells were cultured and treated with MLP for 24 hr. SDS-PAGE and western blotting were used to determine the levels of Cyto-c and Casp-3 protein expression. As shown in Figure 3, MLP significantly increased the induction of Cyto-c and Casp-3 when compared with non-AMP peptides (Figure 3).

***Production of IFN-γ as a primary activation marker for tumor-specific CD8 T cell response***

IFN-γ is a key cytokine in cell-mediated immunity, so we looked at CD8 T cell responses in splenocytes by measuring IFN-production. Two weeks after being treated with MLPs and anti-PD-1 antibodies, the level of IFN- γ secreting CD8 T cells in the spleens of mice treated with MLPs and anti-PD-1 antibodies increased significantly when compared with controls. It is worth noting that the presence of MLP in addition to anti-PD-1 significantly increased antigen-specific T-cell responses (*P*=0.01). Furthermore, mice treated with MLP elicited significant cytotoxic responses against cancer cells, whereas no significant cytotoxicity was observed in the control groups (Figure 4).

***Combined therapy of MPL and anti-PD-1 inhibitors induced the development of systemic antitumor immunity***

To assess the efficacy of antitumor responses, the survival rate of mice and tumor regression was measured (Figure 5). Mice were treated with MLP monotherapies, anti-PD-1 antibody, combination therapy (MLPs plus anti-PD-1 antibody), or PBS after the initial volume of the tumors reached about 65 mm3. Combinational therapy (MLPs plus anti-PD-1 antibodies) significantly reduced tumor volume and growth rate of B10-F16 tumor cells as compared with respective monotherapies. MLPs, anti-PD-1 antibodies, and MLPs plus anti-PD-1 antibodies all showed cytotoxicity on primary tumors and reduced tumor volume (Figure 5a). A Kaplan-Meier survival curve was used to assess the antitumor responses caused by MLPs, anti-PD-1 antibodies, and MLPs plus anti-PD-1 antibodies. In comparison with mice, given MLPs or anti-PD-1 antibodies alone, mice given MLPs plus anti-PD-1 antibodies had the longest survival time. Furthermore, mice treated with MLPs lived longer than mice treated with anti-PD-1 antibodies (Figure 5b).

## Discussion

AMPs are components of living beings’ innate immune systems, and some of them have demonstrated antitumor activity by inducing tumoricidal activity or antitumor immune system activity (16-18). In a previous study, we discovered a new AMP derived from the hippocampus with low cytotoxicity against normal cells, called MLP, and this peptide demonstrated immunomodulatory properties against cancer (15, 18). 

In the present study, we used the orthotropic model for tumor induction, an invasive model of tumor induction, in which cancer cells directly enter the target tissue (19). 

We combined the MLP with an immune checkpoint inhibitor called anti-PD1 antibodies. In previous studies (20, 21), it has been shown that although intratumoral injection of AMPs can prime the response of tumor-specific T-cells; it is because of the presence of immune checkpoints such as PD-L1 or CTLA-4 on the tumor surface, primed specific T cells can be exhausted. Therefore, there is a need to strengthen and support the response of primed tumor-specific T cells. For this reason, we decided to use the intratumoral injection of moronecidin (Piscidin)-like peptide in combination with anti-PD-1 inhibitors to enhance the response of primed tumor-specific T cells and prevent exhaustion by tumor cells.

Several studies have shown that AMPs have cytotoxic effects on cancer cell lines. In line with these findings, Xiaoman *et al*. and Hsiao-Mei *et al*. discovered that AMPs induce apoptosis and necrosis, as well as anti-tumor activity, in various cell lines (20, 21).

Xiaoman *et al*. discovered that Brevivin-1RL1, an AMP, induced caspase-dependent necrosis and apoptosis and could be considered a drug candidate for further development as an anticancer agent (20). Hsiao-Mei *et al*. proposed that MSP-4, an AMP, induced apoptosis in osteosarcoma MG63 cells via an intrinsic and an extrinsic pathway and could be considered a suitable candidate for the treatment of human osteosarcoma (21). Tsung-Lin *et al*. and Jinjing *et al*. also found similar results in their studies (22, 23). 

MLPs’ cytotoxicity to cancer cells can be attributed to the hydrophobic nonpolar face of MLPs’ affinity for the cancer cell membrane (13). After binding to the cell membrane, AMPs form an alpha helix structure through the electrostatic interactions of their hydrophobic part with the plasma membrane, which results in the creation of pores in the cell wall and cell death (24, 25). Previous research has also demonstrated the affinity of AMPs’ nonpolar hydrophobic face to the cancer cell membrane (26, 27). In general, the mechanism of action of AMPs includes cell membrane destruction and endocytosis-mediated penetration into cancer cells, as well as interference with intracellular signaling pathways (12, 21, 28). For this purpose, we examined the effect of MLP peptides on the expression levels of Cyto-c and Casp-3 proteins in B10-F16 cells, which are the most important proteins involved in the induction of apoptosis. MLP significantly increased Cyto-c and Casp-3 induction, which explains MLP’s cytotoxic activity against B10-F16 cancer cells. Similar results have been reported in previous studies with different AMPs (21, 29, 30). 

Harnessing the power of the immune system against tumors is recognized as a promising strategy for preventing tumor recurrence, necessitating the use of immunostimulating agents either alone or in combination with chemotherapy agents, particularly in tumors with a high recurrence rate (31, 32). Recent studies have shown the role of AMPs in modulating the host’s immune system (33, 34). This has caused AMPs to be known not only as antibacterial peptides but also as immunomodulatory mediators (35). Also, sending a dangerous signal from dying cancer cells, inducing chemokine genes, increasing immune responses of T cells, and inhibiting regulatory T cells are other activities and effects of AMPs, which in total increase the potential of anti-tumor immune responses and increase the survival and longevity of patients (36). Thus, we were motivated to search whether MLPs could induce antitumor immunity against clinically murine tumor models and found that treatment with MLPs could induce antitumor immunity and lead to improved survival. This study showed that intratumoral injection of MLPs significantly induced tumor-specific CD8 T-cell response, suggesting the immunostimulatory function of MLPs as described by other AMPs (37-39). 

The ability to stimulate the immune response against the tumor by AMPs, including MLPs, can be assumed to be their advantage compared with chemotherapy.

Here, it was found that intratumoral injection of MLPs improves the ability of anti-PD1 antibodies to induce antitumor immunity. Compared with respective monotherapy, combination therapy with MLPs and anti-PD-1 antibodies elicits a strong antitumor immune response by increasing the number of CD8 + T cells. As confirmed in the current study, the increased IFN- γ secreting CD8 T cells in the spleens of mice treated with MLPs and anti-PD-1 antibodies is the most likely explanation for this finding. 

The antitumor activity and efficacy of several AMPs, including LL-37, P28, and SGX942, have been evaluated in preclinical studies and clinical trials (11, 23, 33, 34).

It has also been proven in previous studies that AMPs can cause the activation of DC cells and antigen cross-presentation, which is another proposed mechanism for stimulating the immune system by AMPs (10, 18). Activation of DCs and antigen cross-presentation plays a key role in inducing antitumor immune responses through the activation of cytotoxic CD8+ T cells (5, 40). In a previous study, we showed that MLPs significantly induced activation markers (CCR7 and CD86) and antigen cross-presentation in bone marrow-derived dendritic cells (BMDCs). In another study, researchers revealed that an AMP named LL-37 up-regulated the expression of the activation markers on the DCs and caused cytotoxic CD8+ T cells to infiltrate tumors and thus tumor regression (10). Therefore, the use of AMPs including MLP can significantly enhance DC immunotherapy protocols.

The efficacy of MLPs is associated with apoptosis (41). Reports have shown that apoptosis induces gene expression and up-regulates chemokines (42). It also activates the release of damage-associated molecular patterns and stimulates innate immunity (43). These facts indicate that apoptosis induced by MLPs induces a strong local immune response and initiates a CD8 + T cell response. Interestingly, although epitope-specific CD8+ T cells were increased in the MLP group in the tumor mouse model, there was no significant increase in the number of surviving mice in this group. We hypothesized that this is due to the abundance of PD-L1+ tumor cells that can block systemic CD8+ T-cell immunity induced by MLP treatment, but in a melanoma tumor model after treatment with MLPs plus anti-PD-1 inhibitors, a significant increase was recorded in survival in mice. In addition to blocking the PD-1 / PD-L1 axis, PD1 inhibitors stimulate the clonal expansion of antigen-specific CD8 + T cells (44). Therefore, CD8 + T cells had a higher proliferative tendency in the MLP plus anti-PD-1 group than in the MLP group. When the PD-1/PD-L1 axis was blocked by an anti-PD-1 inhibitor, an elevated frequency of CD8+ T cells was observed and melanoma tumor remission was observed in group MLP plus anti-PD-1 antibodies. We hypothesized that this is because the ratios of CD8+ T cells to T_regs_ are lower in untreated melanoma tumors than in tumors treated with the MLP plus anti-PD-1 antibodies. 

The present study revealed that intratumoral injection of MLPs plus anti-PD-1 antibodies induced a suitable antitumor response by increasing survival and decreasing tumor growth. This response was a combination of immunotherapy and targeted therapy (combined antitumor response). 

## Conclusion

We used an MLP to improve the anti-tumor response of an anti-PD-1 antibody. Monotherapy with either MLP or the anti-PD-1 antibody demonstrated a low ability to induce an anti-tumor immune response, whereas combination therapy (MLP plus anti-PD-1 antibody) improved anti-tumor immune response, resulting in tumor growth control and increased animal survival. The study provided evidence that intratumoral injection of MLPs could improve the therapeutic effect of intratumorally injected anti-PD-1 antibodies against tumors, particularly in low immunogenic tumors where anti-PD-1 antibodies would have been ineffective.

## Authors’ Contributions

M M and A G designed the experiments; M M, A G, and A M performed experiments and collected data; M M, A G, and A M discussed the results; M M, A G, and AM analyzed and interpreted the results; A G supervised, directed, and managed the study; M M, A G, and A M approved the final version to be published.

## Conflicts of Interest

The authors declare that there were no conflicts of interest.
